# Conserved amino acid networks modulate discrete functional properties in an enzyme superfamily

**DOI:** 10.1038/s41598-017-03298-4

**Published:** 2017-06-09

**Authors:** Chitra Narayanan, Donald Gagné, Kimberly A. Reynolds, Nicolas Doucet

**Affiliations:** 1 INRS – Institut Armand Frappier, Université du Québec, 531 Boulevard des Prairies, Laval, QC H7V 1B7 Canada; 2 Green Center for Systems Biology, UT Southwestern Medical Center, Dallas, TX 75390 USA; 3 PROTEO, the Québec Network for Research on Protein Function, Engineering, and Applications, 1045 Avenue de la Médecine, Université Laval, Québec, QC G1V 0A6 Canada; 4 GRASP, the Groupe de Recherche Axé sur la Structure des Protéines, 3649 Promenade Sir William Osler, McGill University, Montréal, QC H3G 0B1 Canada; 5 Structural Biology Initiative, CUNY Advanced Science Research Center, New York, NY USA

## Abstract

In this work, we applied the sequence-based statistical coupling analysis approach to characterize conserved amino acid networks important for biochemical function in the pancreatic-type ribonuclease (ptRNase) superfamily. This superfamily-wide analysis indicates a decomposition of the RNase tertiary structure into spatially distributed yet physically connected networks of co-evolving amino acids, termed sectors. Comparison of this statistics-based description with new NMR experiments data shows that discrete amino acid networks, termed *sectors*, control the tuning of distinct functional properties in different enzyme homologs. Further, experimental characterization of evolutionarily distant sequences reveals that sequence variation at sector positions can distinguish homologs with a conserved dynamic pattern and optimal catalytic activity from those with altered dynamics and diminished catalytic activities. Taken together, these results provide important insights into the mechanistic design of the ptRNase superfamily, and presents a structural basis for evolutionary tuning of function in functionally diverse enzyme homologs.

## Introduction

The pancreatic-type ribonucleases (ptRNases) are an ideal model system for understanding the relationship between protein structure, dynamics, catalytic function and molecular evolution^1^. Functionally, they constitute a superfamily of endonucleases that catalyze the breakdown of RNAs. Eight catalytically active (canonical) and five catalytically inactive (non-canonical) homologs of RNases were identified in the initial sequencing of the human genome^2^. These homologs, referred to as ‘subtypes’ in the remainder of the manuscript, show roughly 30% sequence identity to one another. Structurally, the characterized canonical RNases share the same kidney-shaped tertiary fold; however, they display a wide range of catalytic efficiencies, differing over five orders of magnitude^3^. Further, these homologous proteins also display a diverse array of biological functions such as angiogenesis, neurotoxicity, antibacterial, antiviral and antihelminthic properties, in addition to the common ribonucleolytic function^2^. This large diversity of biological functions and catalytic efficiencies is an intriguing feature of the rapidly evolving members of the ptRNase superfamily. Biochemical and biophysical characterization of bovine RNase A, an archetypal member of the ptRNase superfamily, and human RNase 3 revealed the role of conformational exchange of distal loops in modulating the catalytic activity of this enzyme^1, 4–6^, providing important insights into factors contributing to the differences in catalytic efficiencies observed for select RNase homologs. However, factors controlling and contributing towards the large diversity in catalytic and biological functions across the superfamily remain largely uncharacterized due to (1) limited or no structural information available for most RNases and (2) lack of a systematic superfamily-wide analysis of diverse sequences to characterize conservation and variation of amino acids across these members. The goal of this study is to define an evolution-based model that can combine the variety of experimental observations across RNase subtypes into a single description consistent with the entire superfamily.

A variety of computational approaches based on amino acid co-evolution have been developed to predict molecular interactions and their role in biological function^7^. One of these approaches is statistical coupling analysis (SCA)^8–10^. The basic premise behind the SCA approach is that amino acid conservation and co-evolution across a set of homologous sequences can be used to infer the pattern of functionally relevant amino acid interactions. This comparative strategy provides us with a single, statistical model for the entire protein family that describes both: (1) invariant sequence features associated with core functions of the family and (2) sequence features associated with the functional divergence of particular subtypes or lineages. First developed by Lockless and Ranganathan^8^, SCA was subsequently applied to a variety of protein families and led to the identification of physically connected, co-evolving residue networks, termed “sectors”, associated with distinct aspects of protein function^9–11^. For example, in the serine protease enzyme family, SCA was used to define three sectors – one associated with core catalytic function, another associated with divergence of catalytic specificity among protease subtypes, and the third associated with protein stability^9^. In a more recent study, Reynolds *et al*. used SCA to identify an amino acid *sector* in the DHFR family whose millisecond conformational dynamics was strongly correlated with enzyme catalysis, consistent with experimental observations, highlighting the evolutionarily conserved nature of residues modulating function^10^.

In this study, we apply SCA to systematically analyze the diverse PtRNase sequences and identify co-evolving amino acid networks that contribute towards the functional divergence of members of this superfamily. Our analysis reveals five subgroups of co-evolving amino acids that relate to distinct aspects of catalysis and dynamics, and that distinguish ptRNase subtypes displaying significant differences in catalytic rates. These five subgroups can be further combined into two quasi-independent *sectors* for this superfamily. Experimental analyses using NMR suggest that these sectors control distinct biochemical functions: sector 1 residues are essential for structural stability while sector 2 residues are largely associated with the ribonucleolytic function. Overall, these results provide a structural model for how variation at specific positions relates to the tuning of function across RNase subtypes, and further provide a general framework for understanding functional diversification in large enzyme families.

## Results

### Identification of co-evolving amino acid networks and functional divergence of ptRNases

**Figure 1:**
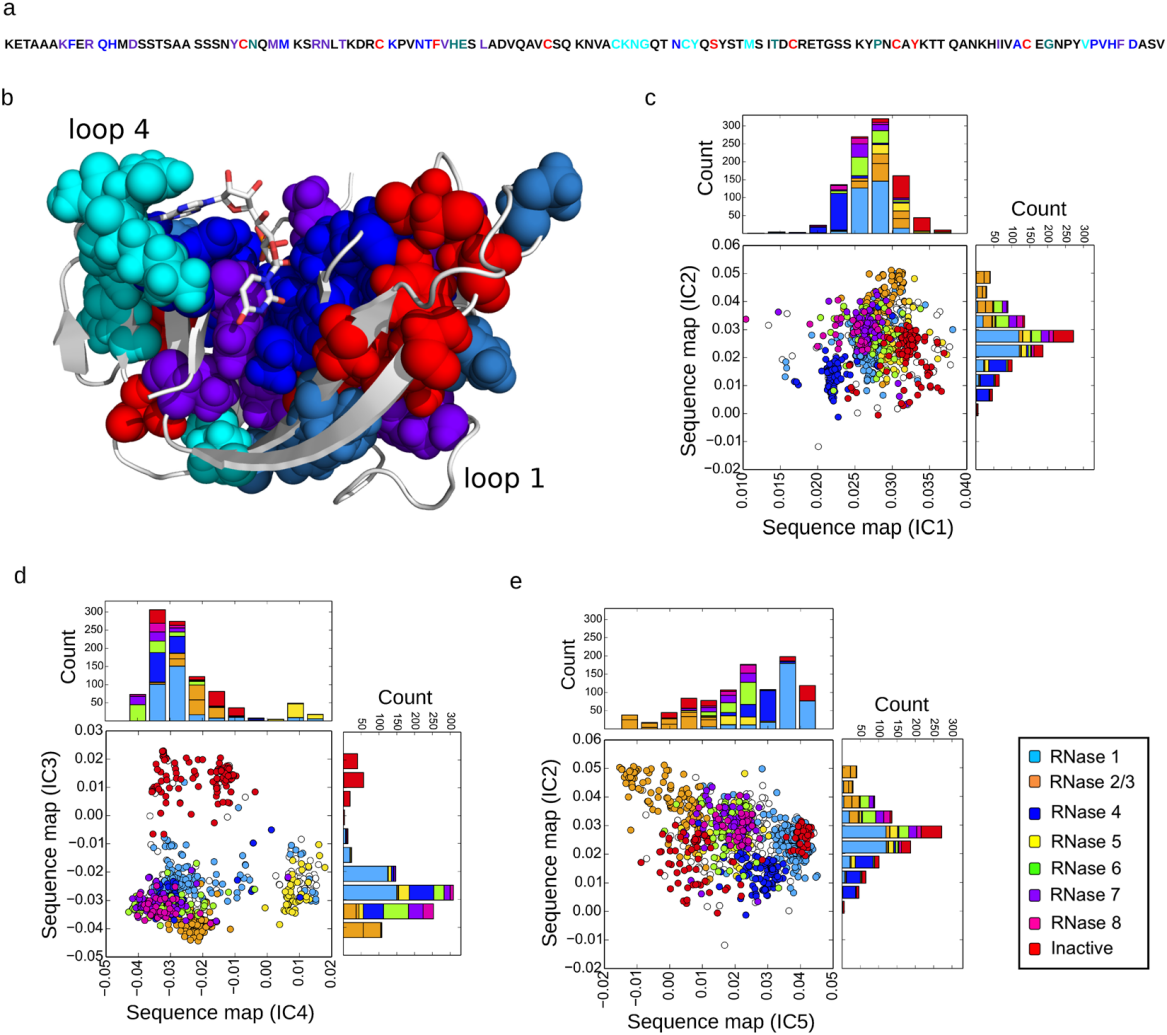
Co-evolving residue networks in the ptRNase superfamily. (**a**) Residue positions of the top five significant eigenmodes (ICs 1–5) are colored red, purple, blue, cyan and teal, respectively, along the primary structure of RNase A. (**b**) Amino acid residues of ICs 1–5, displayed using the space-filling model, mapped on the 3D structure of RNase A (PDB 7RSA). (**c–e**) Each panel shows the scatterplot of sequences (each circle representing a single sequence) along each IC corresponding to sequence variations of positions contributing to each IC. Stacked histograms show the distribution of sequences along each IC. Sequences are colored based on the RNase subtypes found in the entire vertebrate family (RNases 1–8 and inactive members). Sequence distributions along ICs 1 and 2, 3 and 4, 5 and 2 are shown in panels c, d, and e, respectively.

We began by constructing a multiple sequence alignment of 1,922 sequences, spanning all major RNase subtypes (see Methods) to identify networks of co-evolving residues in the ptRNase superfamily. Using the approach described by Rivoire *et al*.^11^, we computed a conservation-weighted correlation matrix (SCA matrix) from this alignment that describes the extent of coevolution between all pairs of amino acids in the protein (Figure S1a). We determined five subgroups of co-evolving amino acid positions, referred to as independent components (ICs) 1–5 from the analysis of the SCA matrix (Figure S1b–d). Like other protein families analyzed to date^9, 10^, we observed that only a subset of positions is strongly co-evolving (48 out of 109 total). While these positions are distributed throughout the amino acid primary structure (Fig. 1a), mapping the five groups of IC residues on the 3D structure of bovine RNase A (PDB ID: 7RSA) shows that they form physically contiguous units in the tertiary structure (Fig. 1b).

To interpret this IC-based decomposition, we used a mathematical mapping method called the singular value decomposition (SVD) to discern the relationship between the five co-evolving ICs and the functional diversification of sequences in the ptRNase superfamily. This approach allows one to relate the pattern of amino acid co-evolution to the functional diversification of sequences in an alignment^11^. Specifically, we link the sequence changes at amino acid positions within a specific IC to the divergence of certain ptRNase subtypes. Figure 1c–e shows the mapping of ptRNase sequences along the five ICs. In the scatterplots, each circle represents a single ptRNase sequence colored based on the annotated RNase subtypes (see Methods). Circles in close proximity along an IC are more similar in the amino acid sequence for the residues constituting the IC, while sequences less similar to each other have evolutionarily diverged and are distinguished by residues of the given IC. For example, in Fig. 1d, we see that the RNase 5 sequences (yellow circles) are separated out along IC4. The interpretation is that the IC4 amino acid positions have undergone correlated evolutionary changes that differentiate the RNase 5 sequences from all other ptRNase subtypes. The histograms along the x- and y-axes provide another representation of this data. They show the distribution of the sequence subtypes along each IC, and further highlight the ability of residues within a particular IC to distinguish different ptRNase subtypes. Overall, this analysis provides an evolution-based model relating small groups of amino acid residues with variations among ptRNase members.

To better understand the functional role of each IC, we conducted a comprehensive study of how residue positions in each IC relate to existing experimental and structural data. The pattern of sequence divergence for IC1 and IC2 are subtler than those for IC3, 4, and 5. Nonetheless, sequence mapping along IC1 shows that residues of IC1 distinguish some of the vertebrate RNase 4 members (Fig. 1c, dark blue) and some catalytically inactive RNase sequences (Fig. 1c, red). Prior mutational studies suggest that positions along IC1 play a critical role in structural stability, with mutations of these residues leading to greater than 20 °C reduction in the melting temperature (Table 1). These include six of the eight cysteines of RNase A, which, together with Phe46, Ser75 and Tyr97, contribute significantly towards stabilizing the protein conformation. In addition to its role in thermal stability, Tyr97 also promotes the correct positioning of the catalytic residue Lys41 through a hydrogen bonding interaction that is conserved in RNase subtypes^12^.

**Table 1:** Functional contribution of sectors defined for the ptRNase superfamily.

IC	Residue mapping on 3D structure	Residue	Function	Reference
1		C26	Stabilizing the conformation (S-S bond with C84)	32
		C40	Stabilizing the conformation (S-S bond with C95)	32
		F46	Chain folding initiation site (CFIS) residue	33
		C58	Stabilizing the conformation (S-S bond with C110)	32
		S75	H-bonding with I106, Hydrophobic core	34
		C84	Stabilizing the conformation (S-S bond with C84)	32
		C95	Stabilizing the conformation (S-S bond with C40)	32
		Y97	CFIS residue (fixes K41 through H-bonding)	12, 35
		C110	Stabilizing the conformation (S-S bond with C58)	32
2		K7	P2 substrate binding site for endonuclease specificity	35
		R10	P2 substrate binding site for endonuclease specificity	35
		D14	H-bonding with Y25	36
		Y25	Hydrophobic core, H-bonding with D14	36
		M29	Hydrophobic core	37, 38
		R33	H-bonding with R10 and M13	39
		N34	Part of N-glycosylation sequence (N34-L35-T36)	40
		T36	Part of N-glycosylation sequence(N34-L35-T36)	40
		V47	Hydrophobic core	38
		L51	Hydrophobic core	38
		I106	Hydrophobic core, expedites folding	34
		F120	Hydrophobic core, fixes the side-chain of H119	34
3		F8	Juxtaposing His12 by π-interaction with His12	35
		Q11	P1 substrate binding site	35
		H12	Catalytic triad residue (General base for catalysis)	34
		M30	Hydrophobic core	38
		K41	Catalytic triad residue (H-bond with transition state)	37
		N44	H-bonding with H12 and K41	34
		T45	Pyrimidine specificity of the B1 site	35
		N71	B2 substrate binding site	35
		A109	CFIS residue	35
		P117	Trans-isomerization for folding	35
		V118	CFIS residue	35
		H119	Catalytic triad residue (General acid for catalysis)	34
		D121	P1 substrate binding site	35

Amino acid residues of each ICs are represented using the space filling model and mapped on the 3D structure of bovine RNase A (PDB 7RSA).

Positions along IC2 separate RNase 4 from RNases 2 and 3 (Fig. 1c, orange) sequences. Top-ranked residues along IC2 correspond to ligand binding sites: Lys7 and Arg10 are involved in substrate recognition and form the P_2_ binding sub-site, while Asn34 and Thr36 are part of the *N*-glycosylation sequence. Other residues of IC2 form hydrophobic core and hydrogen bonding networks (Table 1). Positions along IC3 distinguish RNases 9–13, annotated as catalytically inactive (Fig. 1d, red), from all other canonical sequences. Consistent with this, IC3 residues include the catalytic triad (His12, Lys41 and His119) and residues imparting specificities for substrate binding to the P_1_ (Gln11, Asp121), B_1_ (Thr45) and B_2_ (Asn71) sub-sites in the enzyme. A comparison of residues between the catalytically inactive sequences against all other RNase sequences shows a striking difference - key active site residues including the catalytic triad are either mutated or absent in the catalytically inactive proteins (Table S1). Taken together, these data suggest that both IC2 and IC3 are involved in the core catalytic function of ptRNase homologs.

IC4 residues, localized to the loop 4 region, distinguish RNase 5 sequences (Fig. 1d, yellow) from all other ptRNase sub-types. Comparison of IC4 residues between RNase 5 and other RNases showed a truncated loop 4 in the RNase 5 sequences (Table S1). Shortening of this loop in RNase 5 was previously shown to perturb dynamics and catalytic function, in addition to altering the purine specificity^13, 14^. IC4 therefore describes variation in a loop region that modulates conformational dynamics and activity between the RNase 5 and other ptRNase subtypes. Sequence mapping along IC5 showed the divergence of RNases 2 and 3 (Fig. 1e, orange) from all other ptRNase subtypes. A comparison of the IC5 residues between RNases 2/3 and other ptRNases revealed the replacement of His48 and Thr82 in these sequences (Table S1). We note that RNases 2 and 3 are the only two naturally occurring human RNase subtypes that lack the His48-Thr82 hydrogen bonding interaction that modulates the dynamics of loop 1 and display a corresponding reduction in catalytic activity relative to RNase A and other highly active RNases^15^. The top ranked residues of IC5 (His48 and Thr82) were shown previously using NMR relaxation experiments to form a hydrogen-bonding network (Figure S2) essential for modulating the conformational exchange of loop 1, which is coupled to catalytic activity in the bovine RNase A^5, 6, 16, 17^. Mutation of either residue was shown to result in the loss of conformational exchange^5, 6, 18^ and a corresponding reduction in the catalytic turnover^6^. This shows that variation at IC5 positions can modulate catalytic activity through loop 1 dynamics, and may account for the decreased activity of the RNase 2/3 subtypes.

**Figure 2:**
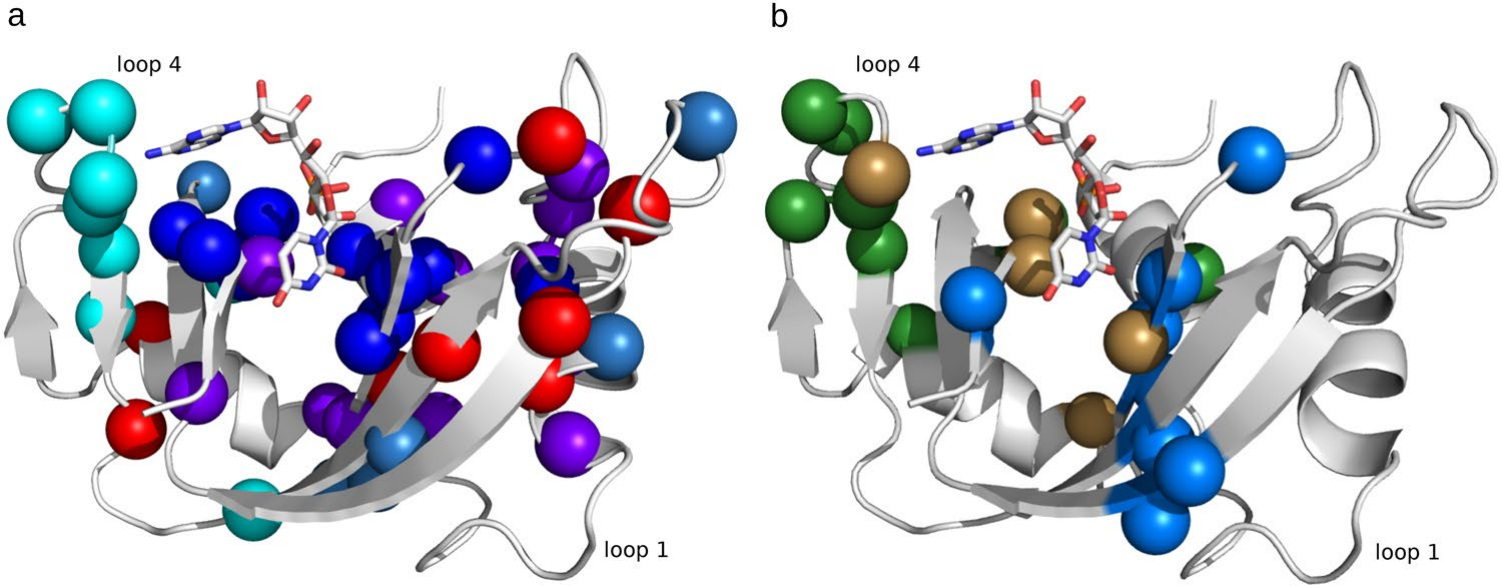
Functional role of co-evolving amino acid networks of ptRNase sectors. (**a**) Amino acid residues of ICs 1–5, displayed as spheres corresponding to Cα atoms, mapped on the 3D structure of RNase A (PDB 7RSA). ICs 1–5 are displayed in red, purple, blue, cyan, and teal spheres, respectively. (**b**) Spheres represent Cα atoms of residues that show NMR chemical shift variations (Δδ) > 0.1 ppm upon incremental titration of RNase A with 5′-AMP (green) and 3′-UMP (marine blue). Brown spheres correspond to residues perturbed by both ligands. Positions of single nucleotide ligands adenosine-5′-monophosphate (5′-AMP) and uridine-3′-monophosphate (3′-UMP), obtained from PDBs 1Z6S and 1O0N, are displayed using stick representations in all Figures. Ligand atoms are colored using the standard coloring scheme – nitrogen, oxygen, carbon and phosphorus as blue, red, white and orange, respectively.

These data provide important insights into the mechanistic *design* of the ptRNase superfamily, and suggest a quasi-modular arrangement in which different biochemical properties that contribute to fitness (stability and activity) have been differentially modulated in evolutionary history. To more carefully test this, we conducted two NMR experiments. First, we performed NMR chemical shift analyses by incrementally titrating two single nucleotide ligands: 5′-AMP and 3′-UMP, up to saturation, with uniformly ^15^N-labeled bovine RNase A, a prototypical member of this ptRNase superfamily. The goal of this experiment was to characterize the involvement of IC residues in ligand binding. We monitored changes in the ^1^H and ^15^N resonance frequencies (Δδ) in the ^1^H-^15^N HSQC spectra for the different ligand concentrations. A total of 14 and 13 residues showed Δδ ≥ 0.1 ppm for the 5′-AMP and 3′-UMP-bound states, respectively, with five residues commonly perturbed by both ligands. Figure 2 shows residues displaying chemical shift variations Δδ ≥ 0.1 ppm for 5′-AMP- (green) and 3′-UMP-bound (marine blue) forms of bovine RNase A. Residues perturbed by ligand binding correspond predominantly to residues of ICs 2–5. Further, residues perturbed by both ligands (brown spheres) were positioned primarily near the transphosphorylation site, corresponding to residues in ICs 2 and 3. This is consistent with the idea that ICs 2 and 3 are critical to the core catalytic function in ptRNases. A list of all residues (and their corresponding Δδ), perturbed by the two ligands is provided in Table S2 of the supporting information.

**Figure 3:**
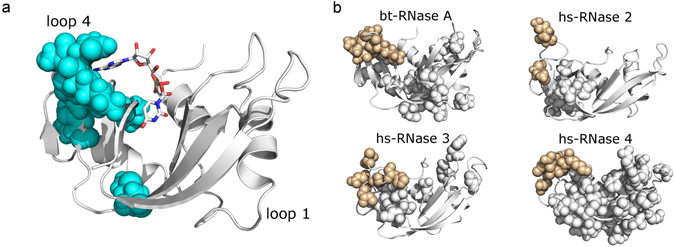
Structural units tuning conformational dynamics. (**a**) Residues of IC4 mapped on the 3D structure of RNase A (PDB 7RSA). (**b**) Millisecond timescale dynamics of free forms of bovine (bt) RNase A (PDB 7RSA), human (hs) RNases 2 (PDB 1GQV), 3 (PDB 1QMT) and 4 (PDB 1RNF), probed using NMR ^15^N-CPMG relaxation dispersion experiments at 500 MHz and 800 MHz and 298 K. Residues showing ^15^N-CPMG dispersion profiles with Δ*R*_*2*_ (1/τ_cp_) > 1.5 s^−1^ are displayed using the space filling representation. Beige color represents residues of loop 4.

While ICs 2 and 3 capture residues that are critical for both 5′-AMP and 3′-UMP binding, positions in ICs 4 and 5 seem to relate to specific ligands. Changes associated with 3′-UMP binding were localized to the β1/β4/β5 regions and includes Thr82, a top ranked residue of IC5 (Fig. 2). In contrast, residues perturbed by 5′-AMP binding were localized to loop 4 of RNase A (Fig. 2a), corresponding to IC4 positions (Fig. 3a), suggesting that residues of IC4 play an important role in purine ligand binding. This measurement is consistent with the observation that variation in IC4 positions distinguish the RNase 5 subtype, and shortening of this loop in RNase 5 alters purine specificity. Further, NMR and MD simulations revealed the highly dynamic nature of this loop^1, 19^ and recent studies showed that the timescale of conformational dynamics in this region coincides with the timescale of catalytic turnover in RNase A^20^, suggesting that the flexibility of this region may play an important role in ligand binding and catalysis.

Observations of functionally important dynamics prompted us to perform NMR ^15^N-CPMG relaxation-dispersion experiments to probe conformational fluctuations on the ms timescale for bovine RNase A and several human RNase subtypes (see Methods). The goal was to examine the conservation of dynamics across subtypes; we expect that loop 4 residues will consistently show conformational exchange, as dynamics in this region is critical for catalysis. Human RNases serve as excellent model systems for probing conserved dynamical properties in the superfamily because of the evolutionarily distant nature of the subtypes that share sequence identities as low as 30%, in contrast to significantly higher identities observed for each subtype across the different taxonomic groups. Fig. 3b shows amino acid residues displaying conformational exchange on the ms timescale using the space filling representation on the three-dimensional structure of each RNase subtype characterized. Our results reveal that conformational exchange of loop 4 residues is observed in all of the characterized RNase subtypes, while significant differences were observed in the global conformational dynamics among the canonical forms of ptRNases. Further, characterization of the dynamics in Macaque and Orangutan RNase 3 homologs showed conserved dynamical properties for the loop 4 region (unpublished data). The only exception to this trend is RNase 5, which shows no significant dynamics on the ms timescale^21^, and is also characterized by a 10^5^-fold reduction in ribonucleolytic activity^3^. These results highlight the conservation of functionally relevant motions of IC4 residues across evolutionarily distant RNase subtypes.

In summary, each of the five ICs determined for ptRNases distinguished the diverse RNase subtypes. ICs 3–5 separated RNase subtypes, in accordance with the functional divergence of these sequences. Specifically, IC3 separated catalytically active RNases from the catalytically inactive forms, while IC4 residues distinguished the RNase 5, characterized by a truncated loop region and a corresponding lower activity and ligand specificity, and IC5 separated subtype sequences (RNases 2 and 3) lacking a conserved hydrogen bonding network shown previously to be involved in the allosteric modulation of catalysis. While the divergence of sequences along ICs 1 and 2 is unclear, mutations in IC1 seem to affect protein stability rather than catalytic activity. None of the ICs distinguished the divergence of sequences based on taxonomic groups, as might be expected for a protein family where multiple subtypes (paralogs) occur within individual species (Figure S3).

### Functional sectors of the ptRNase superfamily

**Figure 4:**
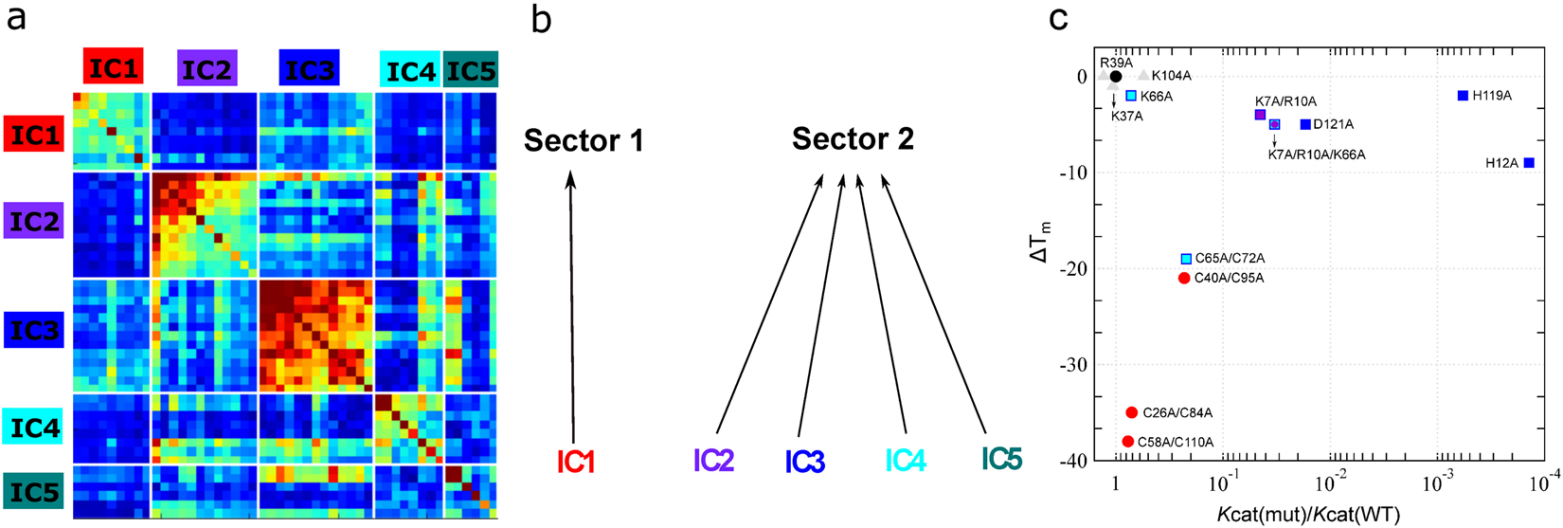
Sector definition for the ptRNase superfamily. (**a**) IC-based sub-matrix of the *C*_*i,j*_ coupling matrix displaying the top five ICs, resulting in the definition of two sectors – sector 1 corresponding to IC1 and sector 2, comprised of ICs 2, 3, 4 and 5. Color scheme of the diagonal elements in the matrix correspond to the intrinsic conservation of residues, with red and blue colors corresponding to high and low conservation, respectively. Colors of the off-diagonal elements reflects the correlation between residues with the red end of the spectrum corresponding to strongly correlated residue pairs while the blue end of the spectrum indicates uncorrelated interactions. (**b**) Two sectors defined based on IC grouping shown in a. (**c**) Effects of amino acid mutations in sectors 1 (red circles) and 2 (squares) on the catalytic rate (*k*_*cat*_) relative to wild type and change in thermal stability (Δ*T*_*m*_ = *T*_*m(mutant)*_ − *T*_*m(WT)*_) in bovine RNase A. The colors of the squares correspond to the IC subgroups defined in Fig. 1. Mutational data were obtained from the literature and are presented for positions where biochemical properties were characterized under the same conditions using polyC as substrate (residues in bold in Table S4). Wild-type data is shown as a black triangle while non-sector residues are displayed as grey triangles.

A comparison of amino acid residues perturbed by ligand binding with those obtained from the SCA sector definition (Fig. 2) showed that ~80% of residues perturbed by binding of the two single nucleotide ligands formed part of the five ICs determined for the ptRNase superfamily. Further, 88% of these residues were localized to ICs 2–5, suggesting that residues along these ICs are likely involved in ligand binding and/or catalysis in the ptRNase superfamily. These observations suggest an architecture of ptRNase enzymes where each IC contributes distinct functional traits that provide the basis for functional variation among RNase subtypes. To characterize and identify functional sectors, we constructed a sub-matrix from the SCA matrix that consists of only positions identified by the ICA based analysis (Fig. 4a). By examining the pattern of inter-IC correlations, we inferred which ICs should be grouped to form sectors. Inter-IC correlations are evident between ICs 2–5, suggesting a common functional role of these IC residues, while IC1 appears more independent. These results indicate two quasi-independent sectors for the ptRNase superfamily – sector 1 composed of IC 1, and sector 2 composed of ICs 2, 3, 4 and 5 (Fig. 4b). We note that this subgroup architecture of sector 2 is not obvious from an examination of the structure or existing experimental data, but only follows from the analysis of sequence statistics across the entire ptRNase superfamily. A list of all sector residues with RNase A sequence numbering is provided in Table S3 of the supporting information.

We also examined the effect of mutations in sectors 1 and 2 on both catalytic turnover and thermal stability by compiling and interpreting discrete prior mutagenesis information from the literature. This compiled data is used here for the purpose of illustrating the effect of mutations in residues that form part of distinct co-evolving amino acid sectors on the catalytic activity and structural stability relative to wild-type. We note that while the biochemical properties of some mutations were characterized under different experimental conditions, a majority of residue replacements analyzed here were investigated under the same experimental conditions by a single research group with the same poly-cytidylate (polyC) substrate. The experimental conditions in which the kinetic and thermal stability parameters were obtained is listed in Table S4. Figure 4c shows the effect of amino acid substitutions on changes in melting temperature and catalytic rate relative to wild type for residues of sector 1 (red circles) and sector 2 (squares) for residues where biochemical properties were determined under similar conditions using polyC as substrate. A comparison of all mutants from the literature is presented in Figure S4. Mutations of sector 2 residues were observed to show large effects on catalytic activity and minimal perturbations on thermal stability, with the exception of the cysteine double mutant (C65A/C72A of IC4). In contrast, mutations of sector 1 residues displayed drastic effects on thermal stability while having a negligible effect on catalytic activity. Mutations of selected non-sector positions (gray triangles) are shown for comparison.

In summary, our observations support the notion that sectors 1 and 2 are evolutionary distinct where the two sector residues quasi-independently control distinct functions, corresponding to structural stability and catalysis. We note that the distinct biochemical properties of the two sectors is not structurally obvious: for instance, positions Cys40 (sector 1) and Lys41 (sector 2) are adjacent in the primary and tertiary structure, yet have profoundly different effects on function. These observations are consistent with previous studies where application of SCA to other protein families demonstrated similar structural organization and independence of biochemical properties^9^. Our results suggest an architecture for ptRNase members where structurally distinct IC subgroups of sector 2 provide the basis for functional variation among RNase subtypes. Specifically, while ICs 2 and 3 directly contribute towards ligand binding and catalytic function, ICs 4 and 5 fine tune the catalytic activity through (1) conformational exchange of IC4 residues shown to be important for ligand binding and catalysis, and (2) modulation of the dynamics of a distal loop important for product release by IC5 residues, respectively.

## Discussion

Our analysis of diverse ptRNase sequences revealed co-evolving amino acid networks, termed sectors, which contribute towards two quasi-independent sectors, consistent with previous observations from other protein families^9–11, 22^. These sectors control two distinct biochemical functions: structural stability and catalytic function. We show that the catalytic sector is composed of sector subgroups, each of which contribute distinct aspects towards modulating the catalytic activity. Sector subgroup positions critical for the core catalytic function are invariant and strongly co-evolving (Fig. 4a), with mutations of these amino acid residues leading to loss of function (Table 1). Not surprisingly, residues of this subgroup distinguish catalytically active and inactive sequences (Fig. 2d). Other subgroups of the catalytic sector are involved in the hierarchical fine-tuning of catalytic function and distinguish functional divergence of specific ptRNase subtypes (Fig. 2d,e). By comparing the dynamical properties of a variety of RNase subtypes across different species on the catalytically relevant ms time frame, we demonstrate the evolutionary conservation of dynamics of a sector subgroup (IC4) across diverse members in this superfamily. These observations emphasize the conservation of amino acid residues that display conformational exchange important for ligand binding and modulation of the ribonucleolytic activity, further illustrating the role of conformational exchange in tuning the enzyme for efficient catalysis and ligand binding.

While the role of structure and dynamics in modulating enzyme function has been characterized for discrete enzyme systems^23^, factors contributing towards the large diversity in catalytic efficiencies and biological function observed within enzyme superfamilies remain poorly understood. Here, we addressed this by systematically analyzing the evolutionarily conserved amino acid networks important for function within the enzyme superfamily using SCA. Previous studies of family-wide sequence analyses revealed protein sectors controlling one or more biochemical function(s)^9–11, 22^. Our results show that sectors are composed of subgroups of amino acid networks that fine-tune catalytic function, and account for the functional divergence observed among sequence subtypes within the enzyme superfamily. These observations emphasize that (1) functional divergence among sequence homologs is rooted in the primary structure and (2) changes in co-evolving sector subgroups impact the functional divergence observed within the superfamily.

Previous studies suggested a correspondence between protein sequence and protein flexibility, and proposed that deviations in this correspondence can provide functional insights^24^. Our results demonstrate that variations in conserved amino acid networks impact the functional variability observed between different members within the superfamily. Observations from this work provide insights into the mechanistic design of the ptRNase superfamily in which functional properties that contribute to fitness, namely structural stability and catalytic activity, have been differently modulated through evolution. Sector residues identified in this work serve as suitable targets for the design of allosteric modulators controlling or perturbing RNase function, and can help guide protein engineering experiments that modulate protein function and enzyme catalysis within specific protein scaffolds. More generally, the approach used in this work provides a framework for analyzing and interpreting the sequence-based data acquired for other enzyme systems.

## Methods

### Statistical coupling analysis (SCA)

Sequences of the ptRNase superfamily were obtained using iterative PSI-BLAST^25^ (e-value cut-off of 0.0001 and a total of nine iterations to convergence) against the non-redundant (nr) database with bovine RNase A as query. Sequence positions were truncated to the structure of bovine RNase A (PDB 7RSA). Multiple sequence alignments were performed using ClustalO^26^. The resulting sequence dataset, consisting of 1,922 sequences and 124 positions, was used for sector definition using SCA. SCA was performed using the python implementation of the software package (pySCA 6.0) (http://systems.swmed.edu-/rr_lab/sca.html). The script annotate_MSA was used to annotate the taxonomic information using the NCBI Entrez utility^27^. Sequences were also annotated based on RNase subtypes from the sequence file headers and were binned into the following categories with keywords used for annotations specified in brackets: RNase 1 (Pancreatic), RNase 2/3 (Eosinophil, Non-secretory), RNase 4 (Ribonuclease 4), RNase 5 (Angiogenin), RNase 6 (Ribonuclease K6), RNase 7 (Ribonuclease 7), RNase 8 (Ribonuclease 8) and RNases 9–13 (Inactive). Sequences that did not fall into the above-mentioned categories were not annotated based on subtype grouping. Pre-processing of the alignment was performed using the scaProcessMSA script to remove gapped sequences (>20% gaps) and those with identities above and below a minimum. A total of 1296 sequences and 109 positions were retained after the pre-processing step. Core calculations and sector definition were performed using the scaCore and scaSectorID scripts. All subsequent calculations were performed according to the documentation accompanying the software package.

### DNA constructs, expression and purification

DNA sequences of RNase A and human RNase 3 (Eosinophil Cationic Protein) were *Escherichia coli* codon-optimized, and subcloned into *Nde*I/*Hind*III-digested expression vector pET22b(+) (EMD Biosciences, San Diego, CA, USA). DNA sequences of human RNase 2 (Eosinophil Derived-Neurotoxin), RNase 4 and RNase 5 (Angiogenin) were acquired from UniProt, *Escherichia coli* codon-optimized, and subcloned into *Nde*I/*Hind*III-digested expression vector pJexpress411/414 (DNA2.0, Menlo Park, CA, USA). For NMR experiments, ^15^N-labeled samples were prepared by growing *E. coli* BL21(DE3) in M9 minimal medium supplemented with non-essential amino acids (Life Technologies, Burlington, ON, Canada), metals, glucose, and ^15^N-labeled ammonium acetate (Sigma-Aldrich, Oakville, ON, Canada). Enzymes were expressed as inclusion bodies and purified as described by Gagné *et al*.^20^. Protein concentration was determined using extinction coefficients of 9,440 M^−1^cm^−1^ (RNase A), 17,460 M^−1^cm^−1^ (RNases 2 & 3), 11,960 M^−1^cm^−1^ (RNase 4) and 11,835 M^−1^cm^−1^ (RNase 5). The extinction coefficients were calculated using ExPASy ProtParam. A purity of >95% was obtained for all proteins, based on SDS-PAGE gel and NMR analysis.

### NMR titration experiments

All NMR titration experiments were performed in 15 mM sodium acetate at pH 5.0. The pH was monitored carefully throughout the experiments and readjusted with sodium hydroxide or hydrochloric acid, when necessary. Single nucleotide ligands 3′-UMP (Chemical Impex Intl Inc., Wood Dale, IL) and 5′-AMP (BioBasic Inc., Markham, ON, Canada) were reconstituted in the same buffer as the protein. ^1^H-^15^N sensitivity-enhanced HSQC experiments were acquired at 800 MHz (18.8 T) using spectral widths (points) of 1600 Hz (256) and 7000 Hz (8192) in the *t*_*1*_ and *t*_*2*_ dimensions, respectively. Binding kinetics was investigated by the titration of each ligand with enzyme/ligand molar ratios of 0, 0.174, 0.393, 0.691, 1.31, 2.71, 6 and 12. Chemical shift differences (Δδ) were calculated as the difference between the free and ligand-bound forms of bovine RNase A according to the following equation^28^:

$${\Delta }\delta (ppm)={[({{(}^{1}H{P}_{0}{-}^{1}H{P}_{sat})}^{2}+{{(}^{15}N{P}_{0}{-}^{15}N{P}_{sat})}^{2}/25)/2]}^{1/2}$$

A cut-off for Δδ was determined as the sum of the average chemical shift perturbation over all amino acid resonances and the standard deviation was calculated to be 0.105 and 0.112 for 5′-AMP and 3′-UMP, respectively.

### ^15^N Carr-Purcell-Meiboom-Gill (CPMG) relaxation dispersion NMR experiments

All NMR experiments were performed at the Quebec/Eastern Canada High Field NMR Facility (QANUC) on Varian (Agilent) 500 (11.7 T) and 800 (18.8 T) MHz NMR spectrometers equipped with triple-resonance cold probes and pulsed field gradients. All proteins were reconstituted at a concentration of 0.4–0.6 mM in 15 mM sodium acetate at pH 5.0 supplemented with 10% D_2_O. Interleaved two-dimensional spectra were collected in a constant time manner with *τ*_cp_ CPMG repetition delays of 0.625, 0.714 (×2), 1.0, 1.25, 1.67, 2.0, 2.50 (×2), 3.33, 5.0, and 10 ms, using a total relaxation period of 40 ms. All NMR spectra were processed using NMRPipe^29^, in-house CPMG scripts and analyzed with Sparky^30^. Global residue fits and model analyses were performed by fitting 500 and 800 MHz CPMG dispersion data to the full single-quantum CPMG equation^31^ using GraphPad Prism 5.

## Electronic supplementary material

Supporting information
