# Supplementary material for: Conserved amino acid networks modulate discrete functional properties in an enzyme superfamily
Source: Sci Rep. 2017 Jun 9;7:3207. doi: 10.1038/s41598-017-03298-4 (PMC5466627; doi:10.1038/s41598-017-03298-4)
Supplement: Supplementary file 1 — Supporting information [file 41598_2017_3298_MOESM1_ESM.pdf]

Supporting information

Conserved amino acid networks modulate discrete functional properties in an enzyme superfamily

Chitra Narayanan[1], Donald Gagné[1,†], Kimberly A. Reynolds[2*], Nicolas Doucet[1,3,4*]

[1]INRS – Institut Armand Frappier, Université du Québec, 531 Boulevard des Prairies, Laval, QC, H7V 1B7, Canada

[2]Green Center for Systems Biology, UT Southwestern Medical Center, Dallas, TX 75390, USA

[3]PROTEO, the Québec Network for Research on Protein Function, Structure, and Applications, 1045 Avenue de la Médecine, Université Laval, Québec, QC, G1V 0A6, Canada

[4]GRASP, the Groupe de Recherche Axé sur la Structure des Protéines, 3649 Promenade Sir William Osler, McGill University, Montréal, QC, H3G 0B1, Canada

*Corresponding authors: nicolas.doucet@iaf.inrs.ca and Kimberly.Reynolds@utsouthwestern.edu

[†]Present address: Structural Biology Initiative, CUNY Advanced Science Research Center, New York, NY, USA

Keywords: Amino acid co-evolution, enzyme dynamics, catalysis, nuclear magnetic resonance, statistical coupling analysis, ribonuclease, millisecond dynamics.

Table S1. Functional divergence of ptRNase subtype sequences along ICs 3, 4 and 5. The 'others' below refer to all RNase sequences other than the specific RNase subtypes being compared to.

IC3	IC4	IC5
RNase1-8: HNMAQTDNHVPKF	RNase5: L----RLF	RNase2/3: RLTSTN
RNase1-8: HNMAQTDNHVPKF	RNase5: L----RLF	RNase2/3: RLTSTS
RNase1-8: HNMAQTDNHVPKF	RNase5: L----RLF	RNase2/3: RLNSTS
RNase1-8: HNMAQTDNHVPKF	RNase5: L----RLF	RNase2/3: RLTSTS
RNase1-8: HNMAQTDNHVPKF	RNase5: L----RLF	RNase2/3: RLTSTS
RNase1-8: HNMA-TDNSVPK-	RNase5: L----RLF	RNase2/3: RLTSTS
RNase1-8: HNMA-THNSVPK-	RNase5: L----RLF	RNase2/3: RLTSTS
RNase1-8: HNMAQTDNHVPKF	RNase5: L----RLF	RNase2/3: RLTSTS
RNase1-8: HNMAQTDNHVPKF	RNase5: L----RLF	RNase2/3: RLTSTS
RNase1-8: HNMAQTDNHVPKF	RNase5: L----RLF	RNase2/3: NTNTTN
Inactive: -HMLLY-YH--PF	Others: CCSNPHVV	Others : HTNTTP
Inactive: -HMVLY-YH--PF	Others: CCSNPHVV	Others : HTNTTP
Inactive: -HMLLY-YH--PF	Others: CCSNPHVV	Others : HTNTTP
Inactive: -HMLLY-YF--PF	Others: CCSNPHVV	Others : HTNTTP
Inactive: -HMLLY-YY--PF	Others: CCSNPHVV	Others : HTNTTP
Inactive: -HMLLY-YH--PF	Others: CCHNPHVV	Others : HTNTTP
Inactive: -HMLLY-YH--PF	Others: CCHNPHVV	Others : HTNTTP
Inactive: -HMLLY-YH--PF	Others: CCSNPHVV	Others : HTNTTP
Inactive: NHITLVQSHVAKL	Others: CCSNPHVV	Others : HTNTTP
Inactive: -HITLV-PN--KF	Others: CCSNPHVV	Others : HTNTTP

Table S2. Amino acid residues of bovine RNase A displaying chemical shift perturbations (Δδ > 0.105 and 0.112 ppm) upon titration with two single nucleotides 5'-AMP and 3'-UMP, respectively.

Residues perturbed by 5'-AMP		Residues perturbed by 3'-UMP		Residues perturbed by 5'-AMP and 3'-UMP
Residue number	Δδ (ppm)	Residue number	Δδ (ppm)	Residue number
Q11	0.127915	H12	0.12682	T45
T45	0.210526	K41	0.163359	L51
L51	0.187528	N44	0.346988	K66
C58	0.215035	T45	0.948202	H119
A64	0.106035	F46	0.210103	F120
C65	0.3676	L51	0.155944	
K66	0.17802	K66	0.1511	
G68	0.113402	T82	0.119922	
Q69	0.12552	T100	0.115356	
N71	0.140585	Q101	0.135389	
C72	0.192262	H119	0.16479	
V118	0.136105	F120	0.397575	
H119	0.221645	A122	0.145866	
F120	0.23242			

Table S3. Amino acid residues constituting the two sectors defined using SCA for the pancreatic-type ribonuclease (ptRNase) superfamily. Sequence numbering is that of bovine RNase A.

Sector	IC	Amino acid residue (order of ranking as in Figure 4a)
1	1	Y97 C95 C84 C40 F46 C58 C110 S75 C26
2	2	K7 Y25 V47 M29 D14 N34 R33 F120 L51 I106 T36 R10
	3	H119 N44 M30 A109 Q11 T45 D121 N71 H12 V118 P117 K41 F8
	4	C72 C65 N67 G68 K66 Y73 V116 M79
	5	H48 T82 N27 P93 E49 G112

Table S4. Summary of catalytic rates and changes in melting temperatures for amino acid substitutions of sector residues in bovine RNase A.

Sector	Residue mutation	ΔT_m (°C)	$k_{cat(mut)}/k_{cat(WT)}$	Experimental conditions	Substrate	Ref.
1 (IC1)	**C26A/C84A**	-35	0.71	10ºC in 0.10 M Mes/NaOH buffer (pH 6.0) containing NaCl (0.10 m), poly(C) 1.18uM-22.7 mM	PolyC	1
	**C40A/C95A**	-21	0.23	10ºC in 0.10 M Mes/NaOH buffer (pH 6.0) containing NaCl (0.10 m), poly(C) 1.18uM-22.7 mM	PolyC	1
	**C58A/C110A**	-38	0.77	10ºC in 0.10 M Mes/NaOH buffer (pH 6.0) containing NaCl (0.10 m), poly(C) 1.18uM-22.7 mM	PolyC	1
	F46A	-22	0.31	25°C in 0.2 M CH3COONa buffer (pH 5.5), with 20 µg/mL enzyme and 0.1-2 mM C>p	C>p	2
	Y97A	-34	0.012	25ºC and 0.10 M MES/HCI buffer (pH 6.0), contain-ning NaCI (0.10 M), PolyC 10uM-0.4mM	PolyC	3
2 (IC2)	**K7A/R10A**	-4	0.045	25°Cin 0.10 M MES/NaOH buffer (pH 6.0) conta-ining NaCl(0.10 M), poly(C) 10uM-1.6 mM	PolyC	4
	F120A	-10.8	0.72	25 °C in 0.2 M CH3COONa buffer (pH 5.5), CpA 0.2-28mM	CpA	5
	Y25F	-6.5	0.8	25°C in 0.1 M acetic acid (pH 5.0), cCMP	cCMP	6
IC2/IC4	**K7A/R10A/K66A**	-5	0.033	25°Cin 0.10 M MES/NaOH buffer (pH 6.0) conta-ining NaCl(0.10 M), poly(C) 10uM-1.6 mM	PolyC	
2 (IC3)	**H12A**	-9	$1.8x10^{-4}$	25°C in 50 mM MES buffer, p H 6.0, containing 0.1 M NaC1, polyC	PolyC	7
	K41G	-9	10^{-4}	25°C, pH 6.0	UpA	8
	A109G	-3	0.25	25°C, pH 6.0	tRNA	9
	**H119A**	-2	$5.8x10^{-4}$	25°C in 50 mM MES buffer, p H 6.0, containing 0.1 M NaC1, polyC	PolyC	7
	**D121A**	-5	0.017	25 °C in 0.10 M MES-NaOH buffer (pH 6.0) containing NaCl, 0.10 M, PolyC 0.05-1 mM	PolyC	10
2 (IC4)	**K66A**	-2	0.72	25°Cin 0.10 M MES-NaOH buffer (pH 6.0) containing NaCl(0.10 M), poly(C) 10uM-1.6 mM	PolyC	4
	**C65A/C72A**	-19	0.22	10ºC in 0.10 M Mes/NaOH buffer (pH 6.0) containing NaCl (0.10 m), poly(C) 1.18uM-22.7 mM	PolyC	1
2 (IC5)	P93A	-11	0.69	22°C and pH 5	cCMP	11
Control	**K37A**	-1	1.063	25°C in 0.10 M MES-NaOH buffer, (pH 6.0), containing NaCl (0.10 M), PolyC 30uM-1.6mM	PolyC	12
	**R39A**	0	1.067	25°C in 0.10 M MES-NaOH buffer, (pH 6.0), containing NaCl (0.10 M), PolyC 30uM-1.6mM	PolyC	12
	**K104A**	0	0.55	25°C in 0.10 M MES-NaOH buffer, (pH 6.0), containing NaCl (0.10 M), PolyC 30uM-1.6mM	PolyC	12

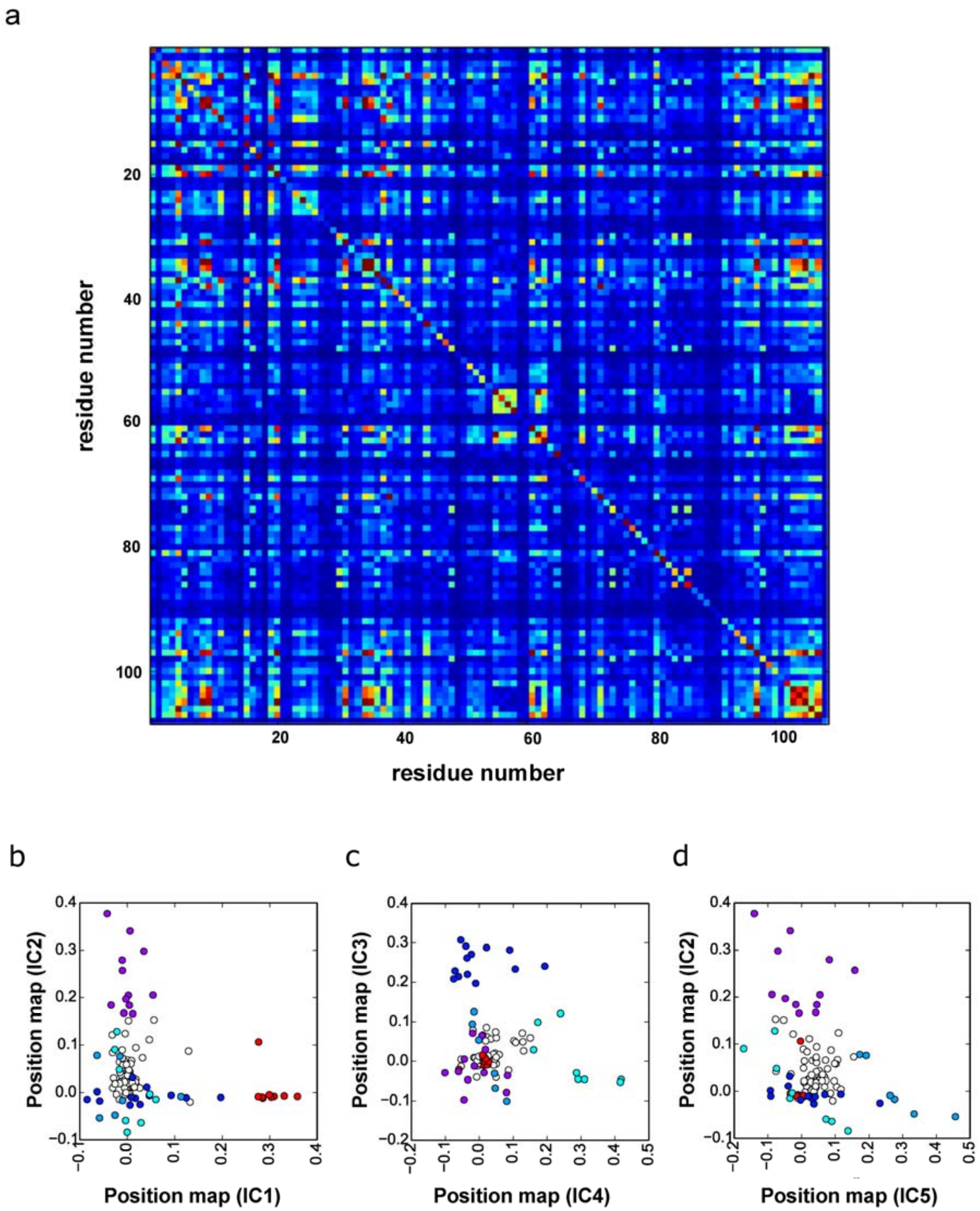

Figure S1. SCA correlation matrix and spectral decomposition for the PtRNase superfamily. a) Positional correlation matrix showing correlations between residues along the primary sequence. b-d) Spectral decomposition of the SCA matrix shows distinct groups of co-evolving residue positions along each independent component (IC). Each circle in the scatter plot corresponds to an amino acid residue represented here using the bovine RNase A primary sequence with amino acids of the different ICs colored as in Figure 1a.

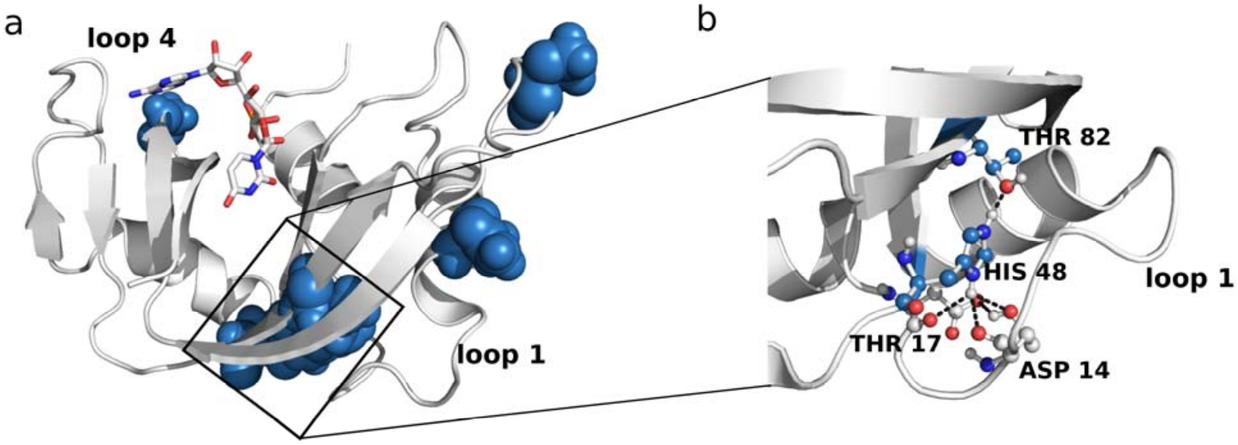

Figure S2. a) Residues of IC5 mapped on the 3D structure of RNase A. b) Zoomed image (of black box in a) of the hydrogen bonding network involving His48 and Thr82 in RNase A. Hydrogen bonds are displayed as dashed lines and atoms of residues presented using the ball and stick model using a standard coloring scheme – blue for nitrogen and red for oxygen. Carbon atoms of residues that are part of IC5 are colored teal while other carbons are colored white. All images were prepared using PyMOL.

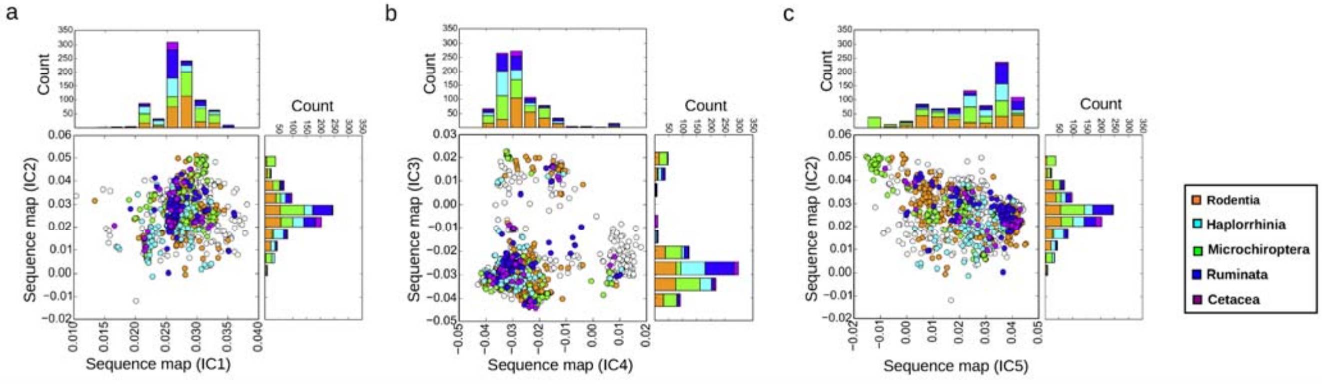

Figure S3. Sequence divergence in the ptRNase superfamily. a-c) Each panel shows the scatterplot of sequences along each IC corresponding to sequence variations of positions contributing to each IC. Stacked histograms show the distribution of sequences along each IC. Sequences are colored based on the taxonomic groups of RNases. Sequence distributions along ICs 1 and 2, 3 and 4, 5 and 2 are shown in panels a, b, and c, respectively.

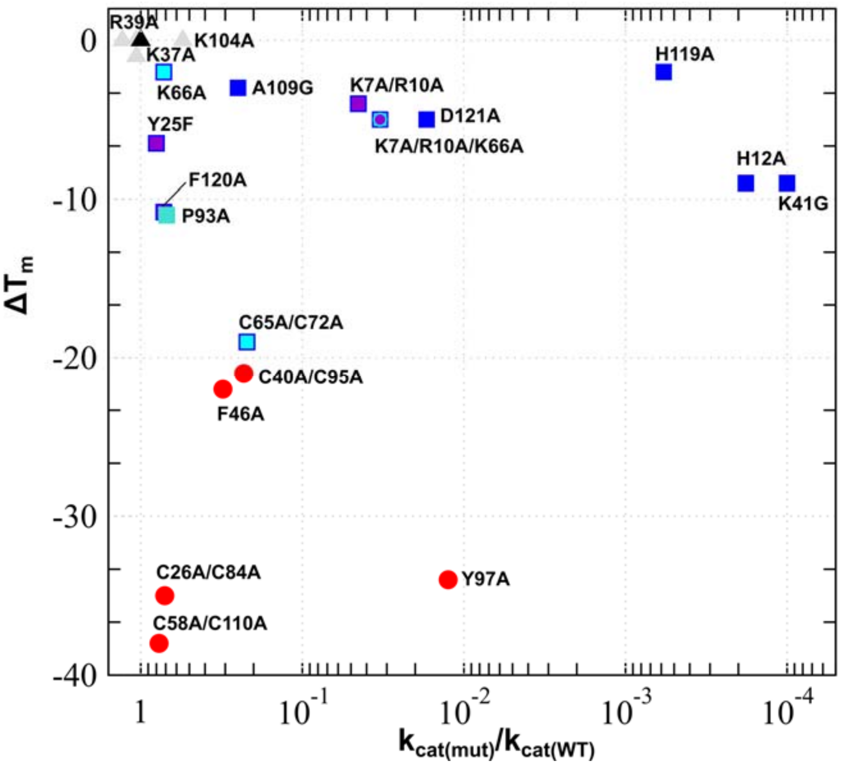

Figure S4. Effects of amino acid mutations in sectors 1 (red circles) and 2 (squares) on the catalytic rate (k_{cat}) relative to wild type and change in thermal stability ($\Delta T_m = T_{m(mutant)} - T_{m(WT)}$) in bovine RNase A. The colors of the squares correspond to the IC subgroups defined in Figure 1. Mutational data were obtained from the literature are presented for all residue positions listed in Table S4. Wild-type data is shown as a black triangle while selected non-sector positions are displayed as grey triangles.

REFERENCES

1. Klink, T.A., Woycechowsky, K.J., Taylor, K.M. & Raines, R.T. Contribution of disulfide bonds to the conformational stability and catalytic activity of ribonuclease A. *Eur J Biochem* **267**, 566-72 (2000).
2. Kadonosono, T., Chatani, E., Hayashi, R., Moriyama, H. & Ueki, T. Minimization of cavity size ensures protein stability and folding: structures of Phe46-replaced bovine pancreatic RNase A. *Biochemistry* **42**, 10651-8 (2003).
3. Eberhardt, E.S., Wittmayer, P.K., Templer, B.M. & Raines, R.T. Contribution of a tyrosine side chain to ribonuclease A catalysis and stability. *Protein Sci* **5**, 1697-703 (1996).
4. Fisher, B.M., Ha, J.H. & Raines, R.T. Coulombic forces in protein-RNA interactions: Binding and cleavage by ribonuclease A and variants at Lys7, Arg10, and Lys66. *Biochemistry* **37**, 12121-12132 (1998).
5. Chatani, E., Tanimizu, N., Ueno, H. & Hayashi, R. Structural and functional changes in bovine pancreatic ribonuclease a by the replacement of Phe120 with other hydrophobic residues. *J Biochem* **129**, 917-22 (2001).
6. Juminaga, D., Wedemeyer, W.J., Garduno-Juarez, R., McDonald, M.A. & Scheraga, H.A. Tyrosyl interactions in the folding and unfolding of bovine pancreatic ribonuclease A: a study of tyrosine-to-phenylalanine mutants. *Biochemistry* **36**, 10131-45 (1997).
7. Thompson, J.E. & Raines, R.T. Value of general Acid-base catalysis to ribonuclease a. *J Am Chem Soc* **116**, 5467-8 (1994).
8. Thompson, J.E. et al. Limits to Catalysis by Ribonuclease A. *Bioorg Chem* **23**, 471-481 (1995).
9. Gagné, D. et al. Perturbation of the conformational dynamics of an active-site loop alters enzyme activity. *Structure*, In Press (2015).
10. Schultz, L.W., Quirk, D.J. & Raines, R.T. His...Asp catalytic dyad of ribonuclease A: structure and function of the wild-type, D121N, and D121A enzymes. *Biochemistry* **37**, 8886-98 (1998).
11. Dodge, R.W. & Scheraga, H.A. Folding and unfolding kinetics of the proline-to-alanine mutants of bovine pancreatic ribonuclease A. *Biochemistry* **35**, 1548-59 (1996).
12. Fisher, B.M., Grilley, J.E. & Raines, R.T. A new remote subsite in ribonuclease A. *J Biol Chem* **273**, 34134-8 (1998).
